# Case of Dislocation of the Os Femoris into the Ischiatic Notch, Successfully Reduced, Ten Weeks and Four Days after the Occurrence of the Accident

**Published:** 1839-10-05

**Authors:** Thomas F. Betton

**Affiliations:** Germantown, Pa.


### MEDICAL EXAMINER.

DEVOTED TO MEDICINE, SURGERY, AND THE COLLATERAL SCIENCES.

No. 40.] PHILADELPHIA, SATURDAY, OCTOBER 5, 1839. [Vol. II.

### CASE OF DISLOCATION OF THE OS FEMORIS INTO THE ISCHIATIC NOTCH, *successfully reduced, ten weeks and four days after the occurrence of the accident.*

By Thomas F. Betton, M. D.

On the 27th of July last, I was requested by my friend, Dr. Egbert, of Manayunk, to visit with him a case of dislocation of the hip-joint, of upwards of ten weeks' standing, in order to consult upon the propriety of attempting its reduction. The case certainly seemed desperate; but, owing to the debility and emaciation of the patient, (a girl, 18 years of age,) consequent on a long and tedious confinement to bed, during the early period of which her life was in danger for several days, and saved only by the skill and care of her physician, she was then in a condition probably more favourable than would ever again occur, and we concluded, after serious deliberation, that it would be proper and safe to make the attempt. Accordingly, on the Thursday following, August 1st, we again met, and proceeded to the reduction.

The patient was placed diagonally upon a hard mattress bed, a counter extending band placed between the pudendum and thigh of the injured side, carried over the shoulder, and secured to the bed-post. The pullies were applied in the usual manner above the knee, secured to the opposite bed-post, and the extension commenced, the leg of the injured side being previously crossed over the sound limb, flexed to a right angle upon the thigh, there to be used as a lever. In about three-quarters of an hour, we had the satisfaction to find the edge of the bone slowly advancing, and rising over the head of the ischiatic notch; by continuing our efforts, it was brought to rest against the acetabulum, on the dorsum of the ilium. Owing to the exhaustion of the patient, it was not considered necessary to use any nauseating or debilitating measures. At this point, Dr. Egbert, standing on the bed directly over the patient, elevated the limb by means of a towel previously passed under the thigh, so as to raise the head of the bone over the edge of the socket. Unfortunately, at this juncture, the rope of the pullies, apparently very strong, gave way; but having provided ourselves against such emergencies, a new rope was soon rove, and, in another quarter of an hour, we had the gratification to find the head of the bone slide into the socket, and the deformity disappear. Owing to the relaxation of the muscles, it caused no noise, such as is heard in the reduction of a recent luxation. A bandage was applied round the pelvis, a solution of morphia ordered, and perfect rest enjoined upon the patient. After the accomplishment of the reduction, the injured limb was found to be somewhat longer than the sound one, owing, as is stated by Sir A. Cooper, to the bone's not entering deeply into the socket.

In the above case, we encountered several difficulties; we found it impossible to keep the band to which the pullies were attached from slipping; the girl's patience became exhausted at the length of time necessary, and the unavoidable severity of the operation, and she begged us, repeatedly, with great earnestness, to desist. To this, however, we did not think it right to accede, and the event justified our perseverance; and now, instead of remaining a cripple for life, she has the full enjoyment of her limbs.

In the London Medical Gazette for July 20th, 1839, will be found an interesting case of a similar accident, complicated, however, with fracture, reduced after the lapse of six weeks.*

*Germantown, Pa.*, October 4, 1839.

* An account of this case will be found in our Foreign Summary. The reporter expresses the belief that there is no other case on record where a successful attempt at reduction had been made after the lapse of six weeks. Dr. Betton's case was of nearly twice that standing.—Eds.

